# Disconjugate Eye Movements in Dyslexic Adolescents While Viewing Op Art: A Creative Handicap?

**DOI:** 10.3390/brainsci12070835

**Published:** 2022-06-26

**Authors:** Lindsey M. Ward, Zoi Kapoula

**Affiliations:** IRIS Laboratory, Neurophysiology of Binocular Motor Control and Vision, CNRS UAR 2022 Neurosciences, UFR Biomedical, University of Paris, 45 Rue des Saints Pères, 75006 Paris, France; lward@mednet.ucla.edu

**Keywords:** saccades, vergence, eye movements, op art, art, dyslexia, creativity, attention

## Abstract

Op art was created, in part, to produce illusions of movement. Given that dyslexics have been shown to have impaired visuo-postural axis deficits, it may be possible that dyslexics see illusions different than their non-dyslexic peers. To test this theory, we measured eye movement and posture in 47 dyslexic (18 female, 29 male; mean age 15.4) and 44 non dyslexic (22 female, 22 male; mean age 14.8) adolescents while they viewed three works of art by Op artist Bridget Riley. They then responded to a questionnaire about how they felt while viewing the artworks. Dyslexics demonstrated significantly slower saccades in terms of average velocity that was particularly disturbed in paintings that manipulated depth. Subjectively, dyslexics felt much more destabilized compared to their peers; however, there was not a significant difference in objective postural measurements between the two groups. The sensation of destabilization was positively correlated with appreciation in non-dyslexic adolescents. These subjective results suggest that dyslexics may be more sensitive to movement in depth, which could be related to the instability in vergence movements. Whereas this instability represents a hinderance in relation to reading, it could be an advantage while viewing paintings such as these.

## 1. Introduction

There are many different ways an artist can invoke motion in a static image: via visual cues such as (dys) symmetry, blurring, forward lean, perspectival representation of pictorial depth, and stroboscopic sequence, to give a few examples [1]. Op art, in particular, creates tension though the organization of lines, form, color, and space to produce many different types of optical illusions, including movement in depth, lateral movement, rotational movement, flickering, hidden images, patterns, and vibrations [2,3].

It has been postulated that the movement sensed while looking at Op art is the product of an autonomous visual response, created by micro eye movements, that is independent of higher visual processing [4]. Specifically, it has been suggested that the illusion of movement is created by small, involuntary microsaccades created by low-level motion detectors induced by the visual form [2,3]. Alternatively, researchers postulate that these motion illusions could be secondary to movements outside of microsaccades, such as slow oculomotor drift or large saccadic eye movements [2,3]. Yet others have described an effect of distractors, or objects that distract or draw the eye towards it, on the dynamics of vergence and saccades movements [5]. Though Op art pieces have never specifically been studied in terms of distractors, there are certainly Op art pieces that have a strong focal piece to which the eye is drawn, with a superimposed illusory motion. In either case, it appears that eye movements across the art form create the illusion of movement in the piece. Previous studies have shown that art, including Op art, can modulate body movement. In one study of 28 healthy adults, two Op art paintings that provide an illusion of depth induced a large antero-posterior body displacement in terms of both speed and space compared with the fixation condition. Such displacements were thought to be secondary to macrosaccades (e.g., saccades with amplitudes larger than 1 degree) occurring under free viewing instructions and also most likely to the small micro-movements, i.e., the vergence drifts during fixations following the macrosaccades [6]. Other artwork that creates pictorial depth cues via perspectival representation has been shown to produce greater postural sway than those without pictorial depth cues [7,8]. 

Outside of depth perception, intentionally arranged motion cues can have a strong impact on a viewer’s posture. One study compared two paintings in which a person was depicted facing towards and away from a strong wind [9]. Viewers were more destabilized in terms of body sway and variance of the speed of body sway, suggesting the intentional depiction of movement in a painting can be translated to the viewer. The neural mechanisms responsible for lateral body sway are sensitive to strength and organization of motion cues in paintings [6,10]. Outside of two-dimensional artistic stimuli, eye movement around three-dimensional objects, such as sculpture, can also induce postural sway [11]. 

There has been a long-recorded history of dyslexics demonstrating abnormal eye movements, both during and independent of reading. Most recently, it has been demonstrated that dyslexics demonstrate peculiar velocity profiles during vergence and saccades. In particular, they exhibit a slow deceleration phase in both vergence and saccades to audiovisual targets, suggesting a slow vergence capacity during both types of movement. During reading, they continue to demonstrate abnormal velocity profiles and have a significantly higher saccade disconjugacy compared with their non-dyslexic counterparts, indicating difficulty in binocular coordination [12]. 

In terms of how dyslexics interact with visual art, they have also been shown to have abnormal eye movements during free exploration of paintings. In particular, dyslexics demonstrated a larger saccade amplitude, as well as a larger conjugate post-saccadic drift, indicating that problems in binocular coordination persist during painting exploration [13].

In addition, the magnocellular theory of dyslexia proposes that there is a deficit in the visual magnocellular system, which is responsible for the timing of vision while reading [14]. If there are unintended eye movements that cause the image to move off the fovea (the so-called “retinal slip”), the magnocellular system is triggered to coordinate eyes back to re-center the target on the fovea, thereby regulating sensitivity to visual motion. It has been proposed that, in the dyslexic population, there is impaired development of the magnocellular layers of the lateral geniculate nucleus, leading to reduced motion sensitivity, impaired binocular fixation, and poor visual localization [15].

Indeed, there have been several experiments that have demonstrated that dyslexics have decreased sensitivity while participating in the coherent motion perception test, a test that helps determine the global perception of motion by evaluating the “motion coherence threshold” [16,17,18]. The lower the number of signal dots required to visualize a moving form, the lower the threshold, and the more sensitive the viewer is to global motion [19]. There has even been one study that has demonstrated that training in improving global motion perception to train magnocellular functioning was associated with improvement in visual errors and reading accuracy, indicating a close link between reading, the visual system, including the magnocellular system, and motion perception [20].

The finding that dyslexics may have decreased motion perception would argue that they may be less sensitive to the motion induced by Op art compared with non-dyslexics. However, given that dyslexics have been shown to have a greater instability of their eye movements, would this instability create an increase in their micro-movements so that they actually see a greater “flicker” effect that would generate a greater contrast and a more sensitive reaction as they survey the painting [12,13,21]? 

Despite the studies that have shown dyslexics preserve their abnormal eye movements independently of reading, the magnocellular theory has been widely debated [22,23]. Typically, dyslexia is considered a primary learning disability. Usually, the typical deficit in dyslexia consists of difficulty with word recognition, decoding, and spelling, which presents as trouble with reading (in terms of decreased comprehension and reading speed) and writing [24,25]. Dyslexia is therefore often considered to be a behavioral disability. Despite many studies that have shown dyslexics demonstrate abnormal oculomotor profiles independent of reading, it is unclear if these abnormal eye movements are the cause or consequence of their difficulty with reading and spelling. Indeed, as others have postulated, dyslexia may not have a single cause, which may make sense given the various presentations that dyslexics exhibit and the complex multisensory integration that is necessary for humans to interact with the world [26]. 

What differences might dyslexics have in terms of posture? Control of posture is related to multisensory input, based on visual, auditory, proprioceptive, and vestibulo-ocular input. Body oscillation causes a slip of the image projected onto the retina, which subsequently provides feedback to the body to provide stabilization; therefore, vision and posture are closely related in a feedback loop in which vision can help stabilize posture [27].

As dyslexics have been shown to have abnormal eye movements, it is a matter of debate whether these abnormal eye movements also contribute to differences in posture. A metanalysis of 17 studies also demonstrated mixed results: balance deficits are associated with dyslexia but may not be uniquely associated with dyslexia [28]. Some have hypothesized that dyslexics suffer from a primary postural deficiency [29,30,31]. Others argue that dyslexics’ postural instability is a consequence of their visual deficits. Some studies have demonstrated that dyslexics exhibit a mild postural instability that can be improved by making specific vergence movements, indicating that dyslexics may suffer from an ocular fixation instability that is tied to an impairment in the communication between vision and postural stability [27,32,33]. Other studies have demonstrated less efficient postural stability compared with non-dyslexics that was uncovered only when challenged with destabilizing prisms and lenses, indicating that the integration of a fragile visual pathway could be at the root of dyslexics’ postural instability [34].

Given these abnormalities in oculomotor movements that persist during movements to targets, reading, and free exploration of paintings, and given dyslexics’ postural abnormalities, which may be related to these abnormal eye movements and integration of sensory stimuli, we posit that dyslexics may explore Op art paintings differently from their peers. As their fragile oculomotor system explores these paintings, they may perceive the motion induced by Op art paintings differently, which may translate into differences in posture.

## 2. Materials and Methods

### 2.1. Participants

A total of 47 dyslexics (18 female, 29 male; aged 10–21; mean age 15.4) and 44 non-dyslexics (22 female, 22 male; aged 8–20; mean age 14.8) were recruited from schools across Paris. The dyslexic adolescents were recruited from a school in Paris that specialized in dyslexic education and were admitted to the school on the basis of their dyslexia diagnosis. The dyslexic adolescents were given their diagnosis at specialized multidisciplinary centers, at which they conducted extensive testing at the time of diagnosis, including neuropsychologic and phonologic testing. From reviewing school records, 34.0% (16/47) identified their primary problem was visual/reading based, for 4.3% (2/47) it was auditory, for 2.1% (1/47) it was writing, and for 59.6 (28/47) it was mixed or unknown. Many of the dyslexic adolescents had co-morbid conditions; twelve were also diagnosed with dysorthographia, dyscalcula, and/or dyspraxia. A total of 34 had been to an orthoptist or were currently enrolled in orthoptic rehabilitation. All participants had no neurologic or psychiatric abnormalities; non-dyslexic adolescents also had no difficulty in reading, vision, or visual impairment. The investigation adhered to the principles of the Declaration of Helsinki and was approved by our Institutional Human Experimentation Committee (CPP CNRS 18 011). Written, informed consent was obtained from the adolescents and/or their parents after they were given an explanation about the experimental procedure. The tests were conducted by two research assistants, who were trained together using the same material and conducted the experiment together for each measurement.

### 2.2. Eye Movement Recording Device

Eye movements were recorded binocularly with a head-mounted video-oculography device, Pupil Core, recording at 200 Hz in binocular vision with an accuracy of 0.60° and a precision of 0.02° (Pupil Labs, Berlin, Germany). 

### 2.3. Accelerometer

Posture was recorded for thirty seconds per condition using a body-fixed sensor, or an accelerometer (Dynaport, MiniMod, McRoberts B.V. The Hague, The Netherlands). The device was placed at L5 on the participant’s lower back using a belt. The MiniMod uses a triaxial seismic acceleration sensor (AXXL202, Analogue Devices, Norwood, MA, USA). The sensor’s full-scale range is +/− 2°. The sampling frequency was set to 100 Hz.

### 2.4. Postural Parameters

The following parameters were analyzed: normalized area (NA in mm^2^/s), root mean square of mediolateral body sway (side-to-side distance or RMS of M/L distance in mm), root mean square of anterior–posterior body sway (forward–backward distance or RMS of A/P distance in mm), root mean square of mediolateral velocity (side-to-side velocity or RMS of M/L velocity in mm/s), RMS of anterior–posterior velocity (forward–backward velocity or RMS of A/P velocity in mm/s), and mean power frequency (MPF in Hz). The first three measurements describe the distance the body moved, whereas the last three describe the energy required to stabilize the body [35]. Body sway with feet placed side-by-side is modulated by two distinct muscle groups: at the ankle for anterior–posterior body sway and at the hip for mediolateral body sway [36,37,38,39].

### 2.5. Calibration of the Pupil Labs Device 

The standard Pupil Labs calibration (Pupil Capture) was applied using a target that was presented at a viewing distance of 1 m. The subject fixated on the center of the target and moved their head rightward, downward, leftward and upward at their own pace. They then repeated the sequence [12].

### 2.6. Procedure

**Figure 1:**
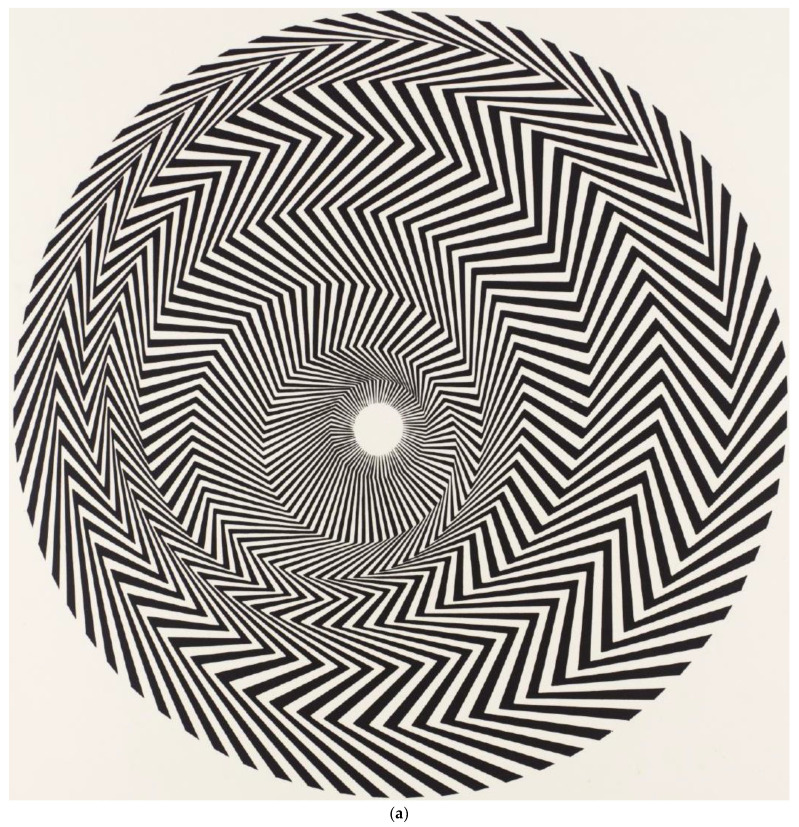

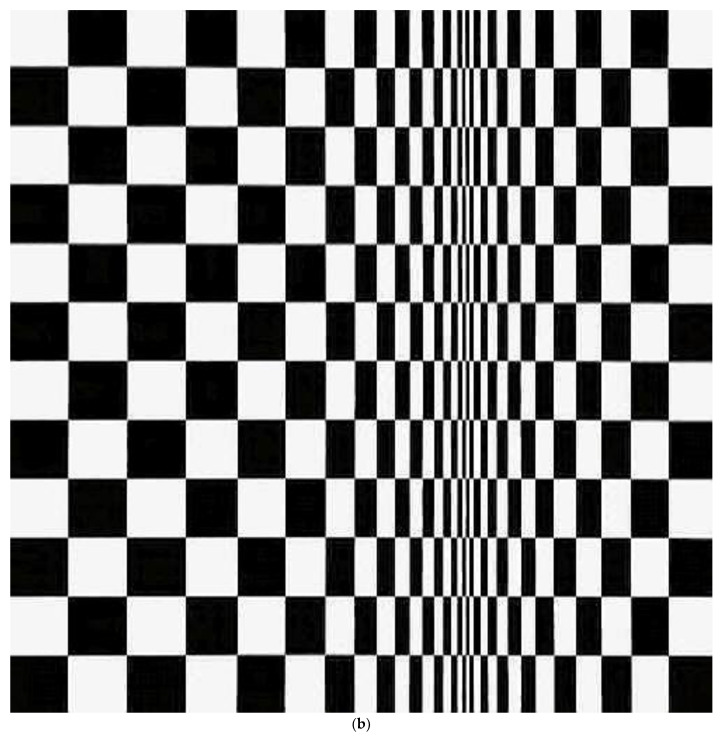

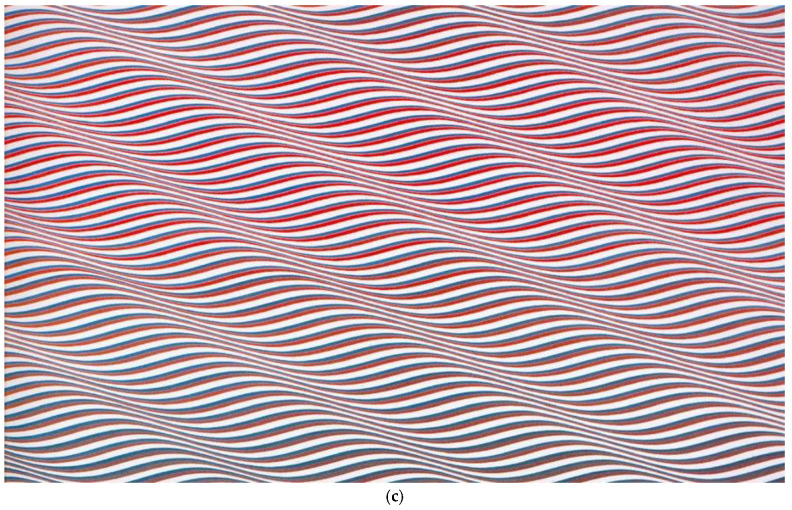
(**a**) Painting 1: Bridget Riley, *Blaze 1*, 1962 © Bridget Riley 2022. All rights reserved. (**b**) Painting 2: Bridget Riley, *Movement in Squares*, 1961 © Bridget Riley 2022. All rights reserved. (**c**) Painting 3: Bridget Riley, *Cataract 3*, 1967 © Bridget Riley 2022. All rights reserved.

Participants were asked to stand in front of a laptop that was positioned 40 cm away from their eyes. The laptop was positioned so that the image would appear in the middle of their vision with the center of the screen located at eye level. The visual angle of viewing each painting was 22.6° in the y-axis. The visual angle in the x-axis was 22.6° for Paintings 1 and 2 and 36.0° in the x-direction for Painting 3. Each adolescent was instructed to try to keep their head still and not to move during the testing. Each participant was asked to fixate at a target on the computer screen at the bottom R corner of the screen prior to being shown each painting. Each participant was invited to explore the image for thirty seconds however they wished when the image appeared. They then were shown each painting on a black background sequentially for 30 s. The same instructions were given to each participant. Eye movements and posture were recorded during each 30 s session. In between each painting they were given 30 s to rest and reset for the next viewing session. They viewed three paintings in a row: Bridget Riley, *Blaze 1*, 1962; Bridget Riley, *Movement in Squares*, 1961; Bridget Riley, *Cataract 3*, 1967. (See Figure 1a–c). 

### 2.7. Paintings

Three paintings were chosen as each gave a different direction of implied movement. The first painting, *Blaze 1*, was chosen as it seemed to give an illusion of circular movement and movement forward in depth. The second painting, *Movement in Squares*, was chosen as it gave an illusion of movement forward in depth and movement from left to right. The third painting, *Cataract 3*, was chosen as it gave a sensation of undulating, wave-like movement from left to right. It was also the only painting that incorporated color, which gives the viewer a flickering sensation due to the contrasting primary colors that are deliberately placed next to one another. All three paintings were chosen because they manipulated depth perception and caused an illusion of the painting moving either in the anterior or posterior plane.

### 2.8. Subjective Evaluation of the Paintings

After viewing the paintings, each child was given a brief questionnaire regarding each of the paintings. While they were filling out the survey, they were given the opportunity to review the paintings on the laptop. They were asked to give a brief written description of what they thought of the painting, how they would rate it on a scale of 1 to 10 (1 = you don’t like it at all; 10 = you love it) and to rank how much they felt destabilized on a scale of 1 to 10 (1 = not at all; 10 = very destabilized). They were also asked in which direction they felt they had moved as they were looking at each painting and were asked in which direction they felt the painting had moved as they viewed it.

### 2.9. Data Analysis

AIDEAL, a software developed in the IRIS laboratory, was used to analyze data recorded with the Pupil Labs eye tracker. To analyze the eye movements, AIDEAL treated the conjugate signal, i.e., the L + R eye position/2. The saccade was defined as the time points where the peak velocity reached above or below 10% of the peak velocity; this corresponded to values above or below 40°/s. AIDEAL defined the average velocity as the ratio of the total amplitude in degrees/time in seconds. The disconjugacy during saccades, or the evaluation of the binocular coordination, were measured as the difference in amplitude between the left and right eye signal. The difference in drift amplitude in the first 80 or 160 ms of fixation was calculated as the disconjugate drift. Eye movements with blinks or artifacts were automatically discarded by AIDEAL. 

### 2.10. Statistical Analysis

The Shapiro–Wilk test was performed for each comparison and all the data was found to be not normally distributed. As such, we performed the non-parametric Mann–Whitney U test for means comparison for eye movements between the dyslexic and non-dyslexic populations as they looked at each painting. We then performed the non-parametric Mann–Whitney U test for means comparison for postural parameters between the dyslexic and non-dyslexic populations as they looked at each painting. In terms of subjective ratings, we performed the non-parametric Mann–Whitney U test for means comparison for subjective ratings of destabilization, appreciation, and of movement between the dyslexic and non-dyslexic populations as they looked at each painting. 

In a second analysis, we calculated the Spearman’s Rho correlation between each of the postural parameters in viewing each painting and the subjective ratings of appreciation, movement, and destabilization. We also calculated the Spearman’s Rho correlation between each of the postural and eye movement parameters in viewing each painting. 

For all analyses, the statistical significance was set at *p* ≤ 0.05. We did not attempt to correct for multiple comparisons. All analyses were performed using SPSS version 25 (IBM Corp. Released 2017. IBM SPSS Statistics for Windows, Version 25.0. IBM Corp.: Armonk, NY, USA).

## 3. Results

### 3.1. Eye Movement Differences between Groups by Painting

For all three paintings, dyslexics demonstrated a higher duration, a faster peak velocity, and a higher average velocity in saccades made to the left and right while viewing (see Table 1, Table 2 and Table 3). This oculomotor profile is similar to that found in dyslexics while reading and while making saccades to audiovisual targets [12,21].

While viewing Painting 1, dyslexics demonstrated a higher disconjugacy during rightward saccades (2.6° +/− 3.1° vs. 1.4° +/− 0.8°; *p* = 0.004). 

For all three paintings, dyslexics demonstrated a lower amplitude while making saccades to the left. While viewing Paintings 1 and 3, they also demonstrated a significantly smaller amplitude (see Table 1a–c). When viewing Painting 2, which had an area of strong visual interest to the right, dyslexics and non-dyslexics did not demonstrate any difference in amplitude in saccades to the right (3.2° +/− SD 0.8° vs. 3.3° +/− 0.1°; *p* = 0.240 respectively). 

While viewing Painting 3, dyslexics demonstrated a higher fixation duration (307.1 ms +/− 90.4 ms vs. 339.2 ms +/− 97.7 ms; *p* = 0.046) and a higher disconjugacy (2.9° +/− 3.6° vs. 1.5° +/− 0.7°; *p* = 0.014) during leftward saccades.

**Figure 2:**
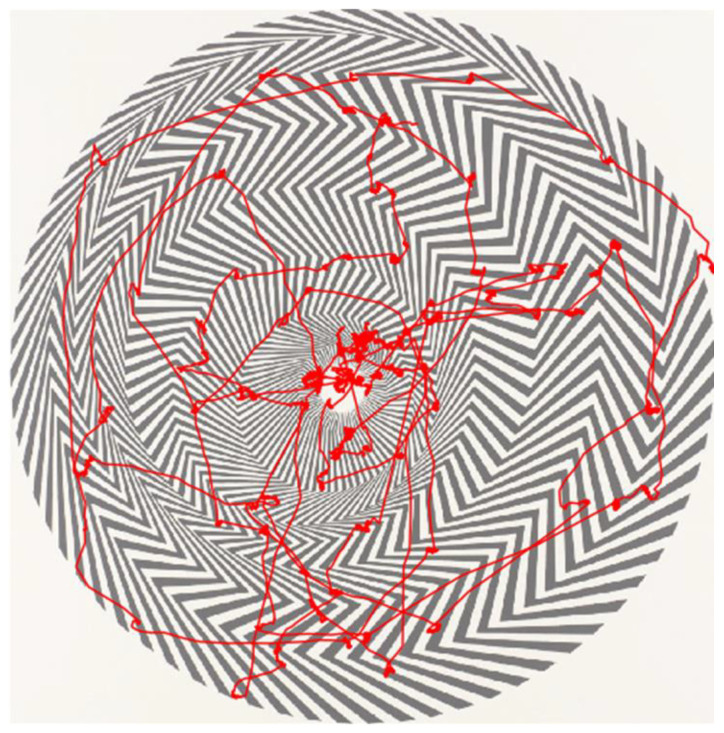
Example of one participant’s eye tracing over the 30 s viewing period.

Please see Figure 2 for an example of one participant’s eye movements during the thirty-second trial overlayed over Painting 1.

### 3.2. Postural Differences between Groups by Painting

**Figure 3:**
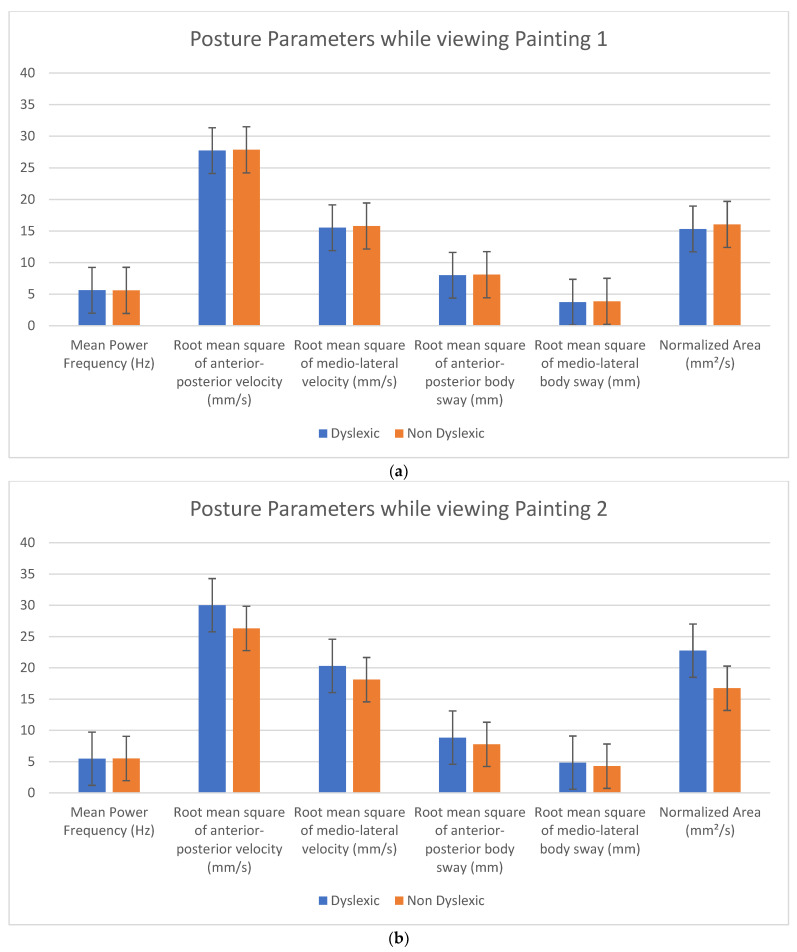

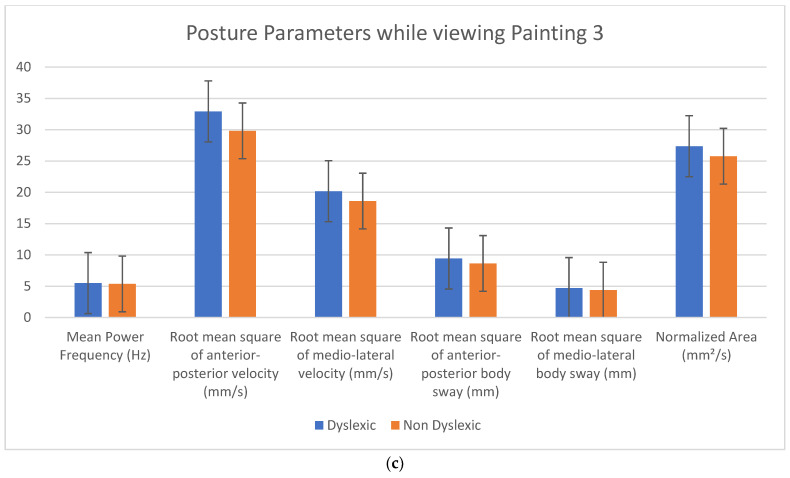
(**a**). Postural parameters in the dyslexic and non-dyslexic populations while viewing Painting 1: Bridget Riley, *Blaze 1*, 1962. (**b**) Postural parameters in the dyslexic and non-dyslexic populations while viewing Painting 2: Bridget Riley, *Movement in Squares*, 1961. (**c**) Postural parameters in the dyslexic and non-dyslexic populations while viewing Painting 3: Bridget Riley, *Cataract 3*, 1967.

There were no significant differences in objective postural parameters measured with the accelerometer device between dyslexic and non-dyslexic children overall and for each individual painting. For all children, for all paintings, there was no difference in objective postural measurements between those who felt some movement and those who felt no movement at all. However, when examining the data for each of the three paintings, it seems that all subjects moved more in the anterior–posterior direction than in the medio-lateral direction (see Figure 3a–c and Supplementary Tables S1–S3). 

### 3.3. Subjective Reports of Destabilization and Movement

Overall, dyslexic children reported feeling significantly more destabilized than non-dyslexic children when comparing reports of destabilization during all three recording sessions (4.7 +/− 3.1 vs. 3.46 +/− 2.8, *p* < 0.001). They also subjectively reported feeling more destabilized for Paintings 1 and 2 (Painting 1: 4.8 +/− 2.9 vs. 3.7 +/− 2.2, *p* = 0.026; Painting 2: 3.7 +/− 2.9 vs. 1.7 +/− 1.8, *p* = 0.001). There was no significant difference between dyslexic and non-dyslexic children in the subjective feeling of destabilization for Painting 3, though dyslexic children reported feeling more destabilized (5.7 +/− 3.1 vs. 5.0 +/− 3.2, *p* = 0.225). 

For Painting 1, dyslexic children subjectively reported that they moved in no direction (14.9% vs. 0.0%) compared with non-dyslexics. Non-dyslexics reported moving in multiple directions (53.2% vs. 71.7%) *p* = 0.050.

For Painting 2, although not significant, dyslexic adolescents reported that they moved more in the left direction (14.9% vs. 4.3%) and in the forward direction (21.3% vs. 8.7%), whereas non-dyslexics reported moving in multiple directions (34.0% vs. 54.3%) *p* = 0.109.

For Painting 3, although not significant, dyslexic children subjectively reported that they moved more in the left direction (12.8% vs. 0.0%) and in the forward direction (12.8% vs. 8.7%), whereas non-dyslexics reported moving in multiple directions (48.9% vs. 65.2%) *p* = 0.184.

For all paintings, there was no difference in the direction children reported for the forms moving.

### 3.4. Appreciation by Group by Painting

**Figure 4:**
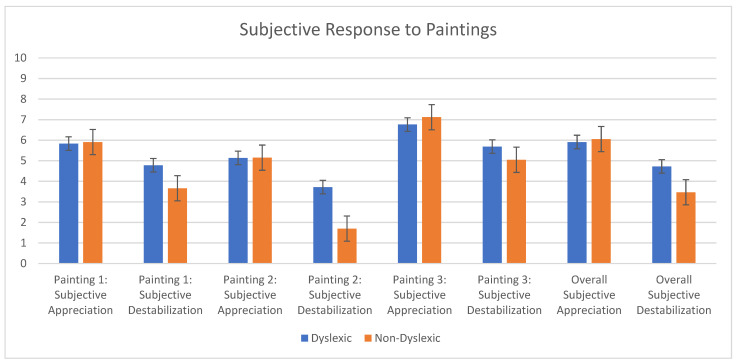
Subjective feelings of appreciation and destabilization after viewing each painting in the dyslexic and non-dyslexic populations.

No significant difference between dyslexic and non-dyslexic children was found in how much they liked the paintings overall or individually per painting, although non-dyslexics reported liking the paintings equally or more than dyslexic children (for all paintings and individually per painting). Please see Figure 4 for a display of subjective appreciation and destabilization by group per painting.

### 3.5. Correlation between Subjective Destabilization and Appreciation by Group

**Figure 5:**
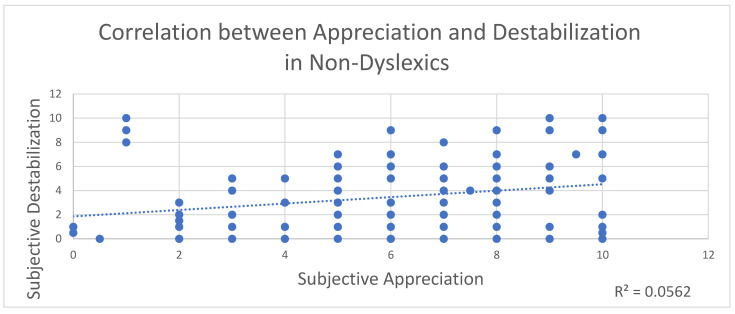
Correlation between appreciation and destabilization for all paintings in non-dyslexics.

For all children, the more destabilized they reported feeling, the more they liked the artwork. This was consistent for all three paintings combined (*p* = 0.005; R^2^ = 0.029). When analyzed by group, the correlation was significant for non-dyslexic children (*p* = 0.006, R^2^ = 0.056; see Figure 5) but was not correlated for dyslexics. Despite the significance, these R values are weak. 

### 3.6. Correlation of Objective Data

There was no correlation between postural parameters and eye movement parameters.

## 4. Discussion

While viewing all three paintings, dyslexics demonstrated uncoordinated eye movements that were similar to those that they made while reading, or while making saccades or vergence movements to audiovisual targets, indicating that their eye movement deficits persist while viewing artwork. Specifically, they demonstrated similar abnormal velocity profiles to those seen while making vergence and saccades to audiovisual targets and while reading, i.e., a faster peak velocity, yet a slower average velocity with a significantly longer duration and a slightly smaller amplitude, suggesting a long deceleration tail that has been discussed in previous studies [12,21]. This observation suggests that abnormalities in the velocity profile of eye movements persist while viewing paintings. Specific to these paintings, dyslexics demonstrated significantly slower saccades in terms of average velocity, which were particularly disturbed in paintings that manipulated depth. From this study, it appears that the dyslexic visual system is disturbed when attempting to coordinate the eyes in depth, even while looking at a two-dimensional painting that is designed to create the illusion of depth.

Despite this unusual visuomotor profile, dyslexics did not demonstrate any difference in how they perceived the illusion moving compared with their peers, showing that there was no difference in how they subjectively interpreted the movement of the illusion. However, anecdotally, dyslexic children reported in their comments after viewing the pieces, particularly Painting 1, that it caused them more pain (headaches, eye pain) to view these optical illusions compared with their peers (see Supplementary Tables S4–S6). This could be attributed to their uncoordinated binocular fixation and the tendency for their eyes to “slip” while attempting to hold fixation. As their gaze does not remain solidly on the target, their eyes make micro-movements to attempt to fixate on a particular target in space. These small oscillations, which attempt to realign their disconjugate gaze, may have triggered an increased subconscious perception of movement, in turn further destabilizing their visuomotor profile; this follows the idea that eye movement instability via micromovements contributes to motion illusion as previously outlined by our laboratory [6]. This hypothesized mechanism is similar to what is known in the clinic as asthenopia and difficulty maintaining fusion in the presence of phoria [40].

Perhaps dyslexics are more sensitive to the optical illusion and visual motion, triggering increased self-generated movements that require increased motor control of the eyes, leading to discomfort while viewing these fluid pieces. The abnormal velocity profiles observed here might be related to abnormalities in efference copy and the differences in perception of visual illusions could be related to inefficient saccadic suppression signals. Testing these hypotheses is unfortunately beyond the scope of this study and needs further research. One way to investigate this would be to study dyslexic and non-dyslexic fMRI while viewing these paintings. By understanding if perceived motion evokes activity in the motion pathways of the brain, one could further delineate how perceived pictorial motion affects the subsequent coordination of eye movement. It is important to note that we chose to present artworks that created illusions in depth, as dyslexics have demonstrated poor binocular fixation, particularly in the depth profile. The fact that dyslexics maintained a discoordinated eye movement profile while viewing these paintings indicates that dyslexics’ eye movements were continued to be perturbed by these Op art forms and this translated subjectively into discomfort.

Interestingly, the eye movement abnormalities that were present while viewing paintings were similar to those present during reading. In comparing these findings with our previous findings in dyslexics while reading, it is important to note that, similarly, dyslexics demonstrate a smaller amplitude, a longer duration, a faster peak velocity, and a slower average velocity while reading and while viewing all three paintings [12,21]. Similarly to reading a difficult text, dyslexics demonstrated a greater disconjugacy while looking at Painting 1, which provokes an illusion of depth. Perhaps the visual conflict that occurs in this perception of depth is similar to the conflict that occurs while attempting to fixate on a difficult text, either resulting in, or because of, difficulties in binocular coordination.

Despite these differences in saccade and vergence profiles, there was no difference in posture parameters between the two groups, indicating that, despite their uncoordinated gaze, dyslexics did not translate this instability throughout the rest of their body. As previously mentioned, there has been some controversy regarding postural control in the dyslexic population. From this study, there is no evidence to suggest that their abnormal visuomotor profile is translated into physical postural instability in the dyslexic population.

Most interestingly, despite there being no physical difference in the direction participants moved, in terms of subjective reports of destabilization and movement, dyslexics reported subjectively feeling more destabilized compared with their peers. Though there was no difference in how each group viewed the forms moving, given their abnormal visuomotor profile, it may be possible that their visual instability may have influenced their sensitivity to visual motion. This contradicts some previous findings that dyslexics have a higher threshold for global motion, though our study focuses more on the illusion of movement in depth compared with previous studies [16,17,18]. Our findings suggest dyslexics may be more sensitive to movement in depth, which could be related to their instability in vergence eye movements. 

Bridget Riley’s work focuses on the manipulation of eye movements to produce an optical response. Her work was demonstrated in a 1965 exhibit at the Modern Museum of Art (MoMA) in New York City entitled “The Responsive Eye”. According to the curator of the exhibition, William Seitz, the works highlighted in the exhibition “exist less as objects to be examined than as generators of perceptual responses…such subjective experiences…are entirely real to the eye, though they do not exist physically in the work itself. Each observer sees and responds somewhat differently” [41].

Indeed, the subjective response created by an objective, physiologic process is what makes Riley’s paintings works of art. While research on dyslexia has focused on deficiencies and inadequacies, exercises such as this one that focuses on subjective interpretation facilitated by different physiology, allows us to focus on dyslexics’ strengths, such as their increased creativity [42]. In French, there is a saying, “le flou artistique”, a colloquialism that translates to “the artistic blur”. Though this study does not examine the relationship between creativity and dyslexic eye movements, perhaps abnormalities in eye movements that persist while viewing artwork, while reading, and while viewing LED targets displayed in the three-dimensional space may give dyslexics a different visual experience that may be aesthetically advantageous. Indeed, Stein has posited that, although dyslexics may have a weaker magnocellular system, they may balance this with a stronger parvocellular system, in which they can better perceive form from background [14]. Perhaps these differences in the visual system can confer disadvantages and advantages according to the situation in which they are perceived. 

There are limitations to our study. We were unable to break-down eye movements while looking at specific portions of the painting; instead, we were only able to take the averages of each eye movement parameters and each postural parameter over the total thirty-second viewing period. We were therefore unable to associate any differences in eye movements between groups that could be associated with different areas of strong visual interest, or focal points in the paintings. We were also unable to compare posture and eye movements while viewing these paintings versus looking at a control image. Future studies should focus on a deeper analysis in which eye movement recording could be synchronized to postural recording sequentially over the course of the viewing period, so that we could better understand how the eye and body are interacting while the viewer focuses on a particular part of the painting. Another possibility would be to define areas of interest in the paintings to examine differences in fixation patterns. Deeper analyses such as these may uncover more subtle interactions of dyslexics and non-dyslexics with artwork. Additionally, a more data-driven approach in the type of oscillations of the body with eye instability may be more revealing. Finally, the differences in amplitude between the two populations, while statistically significant, were small and around the reported spatial accuracy of the eye-tracker. 

## 5. Conclusions

While viewing all three paintings, dyslexics demonstrated discoordinated eye movements that were similar to those they made while reading or while making saccades to audiovisual targets, indicating that their abnormal eye movements persist while viewing artwork. From this study, it seems that dyslexics’ visuomotor system is disturbed when attempting to coordinate the eyes and this results in errors in vergence in depth, even while looking at a two-dimensional painting that is designed to create the illusion of depth. These Op art paintings may have exacerbated dyslexics’ already abnormal visuomotor profile, causing them to feel more destabilized when viewing these pieces compared with their peers. In this way, their discoordinated vision is able to resonate with the message of the artist. Op art exists in the generation of autonomous visual responses and perceptions, rather than an object defined by the canvas; given that they felt more destabilized, perhaps because of their abnormal visuomotor system, dyslexics may be more receptive to these types of works of art and better receptors of the artistic experience. Future studies should consider using art as a stimulus to study the potential creative benefits of observing art. 

## 6. Patents

Zoi Kapoula has applied for patents for the technology used to conduct this experiment: REMOBI table (patent US8851669, WO2011073288); AIDEAL analysis software (EP20306166.8, 7 October 2020; EP20306164.3, 7 October 2020—Europe).

## Figures and Tables

**Table 1:** Eye movements while viewing Painting 1. Bolded values represent statistically significant differences.

	Dyslexic		Non-Dyslexic		*p*-Value	Effect Size
	Median	SD	Median	SD		
**Left Amplitude (deg)**	**3.2**	**0.9**	**4.1**	**1.0**	**<0.001**	**0.45**
**Right Amplitude (deg)**	**3.3**	**0.8**	**4.0**	**1.0**	**0.002**	**0.32**
**Left Duration (ms)**	**82.5**	**48.3**	**62.3**	**30.8**	**0.008**	**0.28**
**Right Duration (ms)**	**89.0**	**79.2**	**59.9**	**38.6**	**<0.001**	**0.39**
**Left Peak Velocity (deg/s)**	**163.6**	**64.7**	**129.9**	**53.8**	**0.003**	**0.31**
**Right Peak Velocity (deg/s)**	**170.7**	**110.0**	**119.6**	**49.4**	**<0.001**	**0.43**
**Left Average Velocity (deg/s)**	**83.5**	**31.1**	**117.8**	**33.9**	**<0.001**	**0.45**
**Right Average Velocity (deg/s)**	**85.8**	**30.0**	**116.7**	**34.6**	**<0.001**	**0.45**
Left Fixation Disconjugacy 80 ms after Saccade (deg)	0.6	0.2	0.7	0.3	0.561	0.06
Right Fixation Disconjugacy 80 ms after Saccade (deg)	0.6	0.3	0.6	0.3	0.781	0.03
Left Fixation Disconjugacy 160 ms after Saccade (deg)	1.0	0.4	0.9	0.4	0.465	0.08
Right Fixation Disconjugacy 160 msec after Saccade (deg)	0.9	0.4	0.9	0.4	0.448	0.08
Left Disconjugacy During Saccade (deg)	2.4	3.0	1.5	0.7	0.086	0.18
**Right Disconjugacy During Saccade (deg)**	**2.6**	**3.1**	**1.4**	**0.8**	**0.004**	**0.30**
Left Fixation Duration (ms)	296.9	68.6	296.3	94.3	0.737	0.04
Right Fixation Duration (ms)	314.1	90.1	316.2	79.1	0.234	0.03

**Table 2:** Eye movements while viewing Painting 2. Bolded values represent statistically significant differences.

	Dyslexic		Non-Dyslexic		*p*-Value	Effect Size
	Median	SD	Median	SD		
**Left Amplitude (deg)**	**3.2**	**0.7**	**3.6**	**1.1**	**0.038**	**0.22**
Right Amplitude (deg)	3.2	0.8	3.3	1.0	0.240	0.13
**Left Duration (ms)**	**86.8**	**38.0**	**74.6**	**66.9**	**0.002**	**0.33**
**Right Duration (ms)**	**87.8**	**50.2**	**64.3**	**40.5**	**0.001**	**0.37**
**Left Peak Velocity (deg/sec)**	**186.9**	**86.5**	**130.1**	**59.6**	**<0.001**	**0.44**
**Right Peak Velocity (deg/sec)**	**176.4**	**80.0**	**130.2**	**78.1**	**<0.001**	**0.41**
**Left Average Velocity (deg/sec)**	**78.0**	**27.2**	**103.5**	**36.5**	**<0.001**	**0.38**
**Right Average Velocity (deg/sec)**	**77.2**	**30.0**	**103.1**	**38.8**	**0.001**	**0.36**
Left Fixation Disconjugacy 80 msec after Saccade (deg)	0.6	0.2	0.6	0.2	0.812	0.03
Right Fixation Disconjugacy 80 msec after Saccade (deg)	0.6	0.3	0.6	0.2	0.266	0.12
Left Fixation Disconjugacy 160 msec after Saccade (deg)	1.0	0.4	0.9	0.3	0.525	0.07
Right Fixation Disconjugacy 160 msec after Saccade (deg)	1.0	0.6	0.8	0.3	0.104	0.17
Left Disconjugacy During Saccade (deg)	3.1	3.8	1.6	1.1	0.063	0.20
Right Disconjugacy During Saccade (deg)	2.7	3.3	1.7	1.7	0.129	0.16
Left Fixation Duration (msec)	306.8	58.2	311.2	95.9	0.674	0.04
Right Fixation Duration (msec)	331.5	83.2	338.2	89.6	0.504	0.07

**Table 3:** Eye movements while viewing Painting 3. Bolded values represent statistically significant differences.

	Dyslexic		Non-Dyslexic		*p*-Value	Effect Size
	Median	SD	Median	SD		
**Left Amplitude (deg)**	**3.5**	**0.9**	**4.2**	**1.1**	**0.001**	**0.33**
**Right Amplitude (deg)**	**3.5**	**0.9**	**4.0**	**1.0**	**0.011**	**0.27**
**Left Duration (ms)**	**88.5**	**54.7**	**58.1**	**20.9**	**<0.001**	**0.40**
**Right Duration (ms)**	**90.3**	**70.0**	**58.5**	**21.3**	**<0.001**	**0.41**
**Left Peak Velocity (deg/s)**	**185.6**	**88.1**	**139.4**	**58.5**	**<0.001**	**0.39**
**Right Peak Velocity (deg/s)**	**188.6**	**80.8**	**135.0**	**56.1**	**<0.001**	**0.45**
**Left Average Velocity (deg/s)**	**92.2**	**38.0**	**132.6**	**40.0**	**<0.001**	**0.45**
**Right Average Velocity (deg/s)**	**91.6**	**33.9**	**128.4**	**37.4**	**<0.001**	**0.45**
Left Fixation Disconjugacy 80 msec after Saccade (deg)	0.7	0.3	0.7	0.3	0.657	0.05
Right Fixation Disconjugacy 80 msec after Saccade (deg)	0.7	0.3	0.7	0.3	0.374	0.09
Left Fixation Disconjugacy 160 msec after Saccade (deg)	1.1	0.5	0.9	0.3	0.520	0.07
Right Fixation Disconjugacy 160 msec after Saccade (deg)	1.0	0.5	1.0	0.4	0.930	0.01
**Left Disconjugacy During Saccade (deg)**	**2.9**	**3.6**	**1.5**	**0.7**	**0.014**	**0.26**
Right Disconjugacy During Saccade (deg)	2.7	3.5	1.4	0.7	0.057	0.20
**Left Fixation Duration (ms)**	**307.1**	**90.4**	**339.2**	**97.7**	**0.046**	**0.21**
Right Fixation Duration (ms)	312.4	79.9	343.6	91.3	0.091	0.18

## Data Availability

The datasets generated during and/or analyzed during the current study are available from the corresponding author on reasonable request.

## Supplementary Materials

The following supporting information can be downloaded at: https://www.mdpi.com/article/10.3390/brainsci12070835/s1, Table S1: Posture parameters while viewing Painting 1; Table S2. Posture parameters while viewing Painting 2; Table S3. Posture parameters while viewing Painting 3; Table S4: Painting 1; Table S5: Painting 2; Table S6: Painting 3.

## References

[1] Cutting J.E. (2002). Representing motion in a static image: Constraints and parallels in art, science, and popular culture. Perception.

[2] Zanker J.M., Walker R. (2004). A new look at Op art: Towards a simple explanation of illusory motion. Naturwissenschaften.

[3] Hermens F., Zanker J. (2012). Looking at Op Art: Gaze stability and motion illusions. Iperception.

[4] Zeki S. (1998). Art and the Brain. Daedalus.

[5] Yaramothu C., Santos E.M., Alvarez T.L. (2018). Effects of visual distractors on vergence eye movements. J. Vis..

[6] Kapoula Z., Lang A., Vernet M., Locher P. (2015). Eye movement instructions modulate motion illusion and body sway with Op Art. Front. Hum. Neurosci..

[7] Kapoula Z., Adenis M.-S., Lê T.-T., Yang Q., Lipede G. (2011). Pictorial depth increases body sway. Psychol. Aesthet. Creat. Arts.

[8] Ward L.M., Morize A., Vernet M., Antoniades C., Kapoula Z. (2021). Art Exists Because the Viewer Exists: How François Morellet’s Tri-ple X Neonly Influences Postural Control and Subjective Aesthetic Appreciation. Leonardo.

[9] Kapoula Z., Gaertner C. (2015). Motion and Lateral Organization in Monet’s Painting Impact Body Sway?. Art Percept..

[10] Gaertner C., Creux C., Espinasse-Berrod M.A., Orssaud C., Dufier J.L., Kapoula Z. (2013). Postural control in nonamblyopic children with early-onset strabismus. Investig. Ophthalmol. Vis. Sci..

[11] Kapoula Z., Lang A., Le T.T., Adenis M.S., Yang Q., Lipede G., Vernet M. (2014). Visiting Richard Serra’s “Promenade” sculpture improves postural control and judgment of subjective visual vertical. Front. Psychol..

[12] Ward L.M., Kapoula Z. (2020). Differential diagnosis of vergence and saccade disorders in dyslexia. Sci. Rep..

[13] Jainta S., Kapoula Z. (2011). Dyslexic children are confronted with unstable binocular fixation while reading. PLoS ONE.

[14] Stein J. (2001). The magnocellular theory of developmental dyslexia. Dyslexia.

[15] Stein J. (2018). What is Developmental Dyslexia?. Brain Sci..

[16] Cornelissen P., Richardson A., Mason A., Fowler S., Stein J. (1995). Contrast sensitivity and coherent motion detection measured at photopic luminance levels in dyslexics and controls. Vis. Res..

[17] Talcott J.B., Hansen P.C., Assoku E.L., Stein J.F. (2000). Visual motion sensitivity in dyslexia: Evidence for temporal and energy integration deficits. Neuropsychologia.

[18] Talcott J.B., Witton C., Hebb G.S., Stoodley C.J., Westwood E.A., France S.J., Hansen P.C., Stein J.F. (2002). On the relationship between dynamic visual and auditory processing and literacy skills; results from a large primary-school study. Dyslexia.

[19] Conlon E.G., Lilleskaret G., Wright C.M., Stuksrud A. (2013). Why do adults with dyslexia have poor global motion sensitivity?. Front. Hum. Neurosci..

[20] Ebrahimi L., Pouretemad H., Khatibi A., Stein J. (2019). Magnocellular Based Visual Motion Training Improves Reading in Persian. Sci. Rep..

[21] Ward L.M., Kapoula Z. (2021). Dyslexics’ Fragile Oculomotor Control Is Further Destabilized by Increased Text Difficulty. Brain Sci..

[22] Blythe H.I., Kirkby J.A., Liversedge S.P. (2018). Comments on: “What Is Developmental Dyslexia?” *Brain Sci*. 2018, *8*, 26. The Relationship between Eye Movements and Reading Difficulties. Brain Sci..

[23] Rayner K. (1985). Do faulty eye movements cause dyslexia?. Dev. Neuropsychol..

[24] Raghuram A., Gowrisankaran S., Swanson E., Zurakowski D., Hunter D.G., Waber D.P. (2018). Frequency of Visual Deficits in Children with Developmental Dyslexia. JAMA Ophthalmol..

[25] Protopapas A., Parrila R. (2018). Is Dyslexia a Brain Disorder?. Brain Sci..

[26] Pernet C., Andersson J., Paulesu E., Demonet J.F. (2009). When all hypotheses are right: A multifocal account of dyslexia. Hum. Brain Mapp..

[27] Kapoula Z., Bucci M.P. (2007). Postural control in dyslexic and non-dyslexic children. J. Neurol..

[28] Rochelle K.S., Talcott J.B. (2006). Impaired balance in developmental dyslexia? A meta-analysis of the contending evidence. J. Child Psychol. Psychiatry.

[29] Quercia P., Seigneuric A., Chariot S., Vernet P., Pozzo T., Bron A., Creuzot-Garcher C., Robichon F. (2005). Ocular proprioception and developmental dyslexia. Sixty clinical observations. J. Français Ophtalmol..

[30] Pozzo T., Vernet P., Creuzot-Garcher C., Robichon F., Bron A., Quercia P. (2006). Static postural control in children with developmental dyslexia. Neurosci. Lett..

[31] Quercia P., Demougeot L., Dos Santos M., Bonnetblanc F. (2011). Integration of proprioceptive signals and attentional capacity during postural control are impaired but subject to improvement in dyslexic children. Exp. Brain Res..

[32] Barela J.A., Sanches M., Lopes A.G., Razuk M., Moraes R. (2011). Use of monocular and binocular visual cues for postural control in children. J. Vis..

[33] Barela J.A., Tesima N., Amaral V.D.S., Figueiredo G.A., Barela A.M.F. (2020). Visually guided eye movements reduce postural sway in dyslexic children. Neurosci. Lett..

[34] Kapoula Z., Gaertner C., Matheron E. (2012). Spherical lenses and prisms lead to postural instability in both dyslexic and non dyslexic adolescents. PLoS ONE.

[35] Vernet M., Morize A., Kapoula Z., Kapoula Z., Volle E., Renoult J., Andreatta M. (2018). Postural and Emotional Impact of Carsten Höller’s Artwork “Light Corner”. Exploring Transdisciplinarity in Art and Sciences.

[36] Gatev P., Thomas S., Kepple T., Hallett M. (1999). Feedforward ankle strategy of balance during quiet stance in adults. J. Physiol..

[37] Winter D.A., Prince F., Frank J.S., Powell C., Zabjek K.F. (1996). Unified theory regarding A/P and M/L balance in quiet stance. J. Neurophysiol..

[38] Winter D.A., Patla A.E., Ishac M., Gage W.H. (2003). Motor mechanisms of balance during quiet standing. J. Electromyogr. Kinesiol..

[39] Day B.L., Steiger M.J., Thompson P.D., Marsden C.D. (1993). Effect of vision and stance width on human body motion when standing: Implications for afferent control of lateral sway. J. Physiol..

[40] Abdi S., Rydberg A. (2005). Asthenopia in schoolchildren, orthoptic and ophthalmological findings and treatment. Doc. Ophthalmol..

[41] Seitz W.C., Museum of Modern Art (1965). The Responsive Eye.

[42] Kapoula Z., Ruiz S., Spector L., Mocorovi M., Gaertner C., Quilici C., Vernet M. (2016). Education Influences Creativity in Dyslexic and Non-Dyslexic Children and Teenagers. PLoS ONE.

