# Supplementary material for: Disconjugate Eye Movements in Dyslexic Adolescents While Viewing Op Art: A Creative Handicap?
Source: Brain Sci. 2022 Jun 26;12(7):835. doi: 10.3390/brainsci12070835 (PMC9312852; doi:10.3390/brainsci12070835)
Supplement: Supplementary file 1 [file brainsci-12-00835-s001.zip › brainsci-1699498-supplementary.pdf]

Supplementary Materials: Subjective data
Posture Data

Table S1. Posture parameters while viewing Painting 1.

	Dyslexic Median	SD	Non-Dyslexic Median	SD	P-Value
Mean Power Frequency (Hz)	5.6	1.8	5.6	1.7	0.43
Root mean square of anterior-posterior velocity (mm/s)	27.7	25.7	27.9	25.4	0.23
Root mean square of medio-lateral velocity (mm/s)	15.5	10.4	15.8	10.5	0.65
Root mean square of anterior-posterior body sway (mm)	8.0	8.9	8.1	8.8	0.25
Root mean square of medio-lateral body sway (mm)	3.7	2.7	3.9	2.8	0.72
Normalized Area (mm^2/s)	15.3	25.8	16.0	26.2	0.45

Table S2. Posture parameters while viewing Painting 2.

	Dyslexic Median	SD	Non-Dyslexic Median	SD	P-Value
Mean Power Frequency (Hz)	5.5	1.7	5.5	1.6	0.73
Root mean square of anterior-posterior velocity (mm/s)	30.0	23.5	26.3	15.3	0.29
Root mean square of medio-lateral velocity (mm/s)	20.3	14.8	18.1	12.0	0.10
Root mean square of anterior-posterior body sway (mm)	8.8	7.8	7.8	5.0	0.63
Root mean square of medio-lateral body sway (mm)	4.8	4.5	4.3	4.0	0.11
Normalized Area (mm^2/s)	22.7	41.0	16.7	29.9	0.36

Table S3. Posture parameters while viewing Painting 3.

	Dyslexic Median	SD	Non-Dyslexic Median	SD	P-Value
Mean Power Frequency (Hz)	5.5	1.9	5.4	1.8	0.99
Root mean square of anterior-posterior velocity (mm/s)	32.9	29.5	29.8	26.8	0.35
Root mean square of medio-lateral velocity (mm/s)	20.2	15.6	18.6	13.5	0.30
Root mean square of anterior-posterior body sway (mm)	9.4	9.3	8.6	9.1	0.86
Root mean square of medio-lateral body sway (mm)	4.7	4.9	4.4	4.5	0.73

Normalized Area (mm^2/s)	27.4	68.2	25.8	76.8	0.89

Table S4: Painting 1

Dyslexic	Subjective Appreciation	Subjective Destabilization	What do you think of the painting?
Y	5	3	There are geometric forms
Y	10	5	I find it beautiful
Y	3	2	I don't think it's pretty
Y	1	1	**This paintings hurts my head**
Y	8	5	**This painting grabs you and hurts my eyes. I like paintings like this that look like they are moving**
Y	4	8	**This painting is weird and it hurts my eyes**
Y	2	1	**It hurts my eyes**
Y	3	5	When you fixate just below the painting you get the idea it's moving
Y	7	6	This painting makes me think of a filter, with stairs around the sides. It gives you the impression of emptiness
Y	4	6	Not very interesting
Y	5	6	Like a shell, makes you think of a funnel made of multiple different levels
Y	7	10	This painting is good
Y	10	1	This painting makes me think of a telescope
Y	10	8	I really like this painting because it gives a funny optical effect.
Y	6	1	I don't like it
Y	7	8	It is pretty
Y	7	0	I like it a lot
Y	8	7	**This painting makes my eyes tired. It makes my eyes turn around.**
Y	1	6	Nothing, normal.

Y	5	4	**This painting makes my head hurt if I look at it too long.**
Y	7	2	I think it's good and beauitful.
Y	10	10	It is pretty this large painting with two colors: white and grey
Y	7	2	A circle
Y	9	3	It gives me a reassuring and calm effect
Y	5	5	The painting sometimes turns and sometimes is normal
Y	4	6	For me it's a neverending trace
Y	7	6	I think this painting makes me think of a tapestry that changes direction with each level and circle
Y	10	5	I see a circle like a funnel with lines that aren't very straight, like in a column
Y	10	9	It's okay, it's beautiful
Y	6	9	It envelopes you and hypnotizes
Y	3	5	I think it makes us think of infinity with the spirales
Y	5	10	It's like a whirlwind
Y	3	7	**I think it's disturbing because it destabilizes my eyes**
Y	10	1	It's a hypnotizing painting
Y	3	6	I think this painting is a spiral without an end which finishes with a hole
Y	7	6	It has a vibratory effect, like a film. Sometimes it moves towards the center, then towards the outside. The sides move either towards the left or towards the right.
Y	3	4	First of all, this painting is an optical illusion: it draws the eye towards the center of the image.
Y	4.25	1	This painting is based on the visual effect of an optical illusion and on the impression of movement of the fixed image. But this movement is from the 15th century, it's not original.
Y	7	2	It seems like a tunnel or a donut.

Y	8	1	It seems like a hole and it's well done.
Y	4	3	It's a circle that's an optical illusion.
Y	5	2	It's an illustration that will never stop.
Y	0	0	**It hurts me**
Y	9	5	It's round with hypnotic patterns. When you focus on it it moves around
Y	4	2	it is a black and white spiral. It is an optical illusion
Y	5	7.5	I think I feel dizzy
Y	6	9	The painting is interesting because its simple line work and creates a deep and inescapable feeling of depth. The painting is abrasive to the eyes because they almost work against your mind because you know what you should see but you aren't
Y	6	8	Interesting in that when you don't fully focus it seems to move
N	4	3	It looks like it's moving
N	7	5	I think this painting is troubling, because I see movement even when there isn't any.
N	9	1	It seems blurry
N	5	3	It looks like a round turret or a volcano
N	5	5	Spiral black and white
N	7	5	It's almost like you're falling deeper and deeper into the painting. You can see the lines moving in a circle sometimes they look like lines and sometimes like triangles
N	5	7	I think that this painting is an optical illusion and I think that it moves like the circle are turning
N	8	4	Illusional, slight spinnings can be noticed
N	7	2	Very simple but hypnotizing in a fun way. It's a sort game where you have to follow the arrows in my opinion
N	6	1	The painting is interesting, it moves everywhere except where I'm looking, or the more I move my eyes, the more it moves.

N	3	2	Annoying circles that want to move. And ziggzags
N	8	6	The painting made me feel like I could fall into it
N	8	6	It's an optical illusion in black and white in the shape of a spiral. The spiral closes itself before clsoing up leaving a white spot. The lines are shaped in a sort of arrow and form a circle.
N	7	2	It makes me want to fall asleep because each of the black lines looks like a bunch of Zs.
N	1	9	It's very crazy and circular. It's also like a maze but for me when I started it I didn't see the inner white circle in the moving
N	7	4	Very movemented, the definition between the circles is very interesting, I like the pattern
N	5	7	The painting very beautiful and crazy
N	10	2	It looks circular and your eyes can follow the spin. I think that it's a good painting because kind of like a roler coaster
N	7	2	tunnel effect. I find it interesting, it seems to me that there are some sort of steps.
N	7	5	It's ok. I understand how it was made so it is less mesmorizing.
N	6	2	It's interesting and looks a bit like a swirling eye with eyebrow stripes
N	7	8	Whirlpool, contrast, dizzy
N	5	6	It's weird and tricks the eye. It looks like a vortex
N	4	3	There is an interesting use of positive and negative space. I like the illusions because the impression of different shapes can be seen; circles, lines, and triangular shapes. The triangular shapes seem 3D like pyramids but from an above-view.

N	7	3	I think it's a spiral image and tha tit goes on forever, but it is hard to find the end of the painting.
N	3	5	It's round. A bit messy
N	2	1.5	It is pretty boring. If you just follow the lines slowly, there is no movement. Nevermind, now, I see no movement at all.
N	7	6	It seems like it moves. It's bizarre and concentrates on a focal point
N	5	6	I don't like it very much because it is strange to look at something that gives the impression that moves
N	8	0	It looks like an illusion because you can see it moving but its not moving. It was well done for what its meant to be
N	5	7	The painting is quite tricky because of the lines that go in many different directions
N	8	4	When looking at the painting it makes you want to follow the lines and also try to look for patterns
N	7	3	A shape is spherical. It doesn't hur the eye unless you focus your vision
N	8	1	See it turning, going around in circular movements
N	9	4	I think the painting is really helpful for one's concentration, and has a lot of depth to it, you can also see this painting from different perspectives. I personally can imagine the painting being absolautely flat and also imagine it being a hole
N	5	2	Nothing special
N	5	3	I find the painting odd as it looks like a spiral but on closer inspection is really a bunch of rings they also appear to move
N	2	0	It looks a bit like the kalaedescopes I used to play with as a kid. It looks like an artist's

			interpretation of infinity as the spiral continues into a minute scale
N	3	1	This painting looks like a liquorish roll
N	4	3	I like how every row has a different pattern and that it goes into nothing
N	6	2	I quite like the painting because the artist managed to make a 2 dimensional surface look like something three dimensional with depth, the illusion is very interesting but I think it lacks emotion
N	8	3	I think that the painting starts to move like some kind of animated computer picture. It is a little bit like an optical illusion, hypnotizing
N	4	3	Very beautiful painting, makes you think of a tunnel.

Table S5: Painting 2

Dyslexic	Subjective Appreciation	Subjective Destabilization	What do you think of the painting?
Y	4	3	It's original, you could say there's a break on the right
Y	10	5	I find it beautiful
Y	4	2	It's okay, it's very precise
Y	7	1	It's original
Y	6	4	**It hurts my eyes**
Y	5	7	Bizarre
Y	5	1	**There is an optical effect but it hurts my eyes**
Y	3	2	Looks like a chessboard
Y	8	8	It seems like the painting gets closer and then pulls away
Y	4	4	Not very original
Y	3	5	While looking at this painting, it looks like two rollers that barely touch but turn in towards each other
Y	10	0	This painting is too cool
Y	6	0	It looks like a checkerboard that implodes onto itself
Y	9	8	I really like this painting a lot. It makes me think of a chessboard on a white sheet that folds in on itself
Y	7	3	I don't like it
Y	6	6	It looks like curtains
Y	7	0	I like it
Y	4	3	It looks like the center of a book
Y	1	4	Nothing
Y	3	3	The painting is fine, even if it doesn't make me want to look at it because it's so ordinary
Y	7	5	It's pretty with the squares
Y	1	10	I saw this table move to the left
Y	4	2	A checkerboard
Y	4	3	It makes me think of a chessboard
Y	3	0	It's lame
Y	6	1	It's a flat platform that falls deeper and deeper

Y	5	6	I think it makes me think of a sink because the squares get smaller and smaller
Y	10	2	I see that there is a square in the middle with more and more smaller squares surrounding them. There is also a line that separates the smaller squares
Y	9	9	It is pretty
Y	7	10	Hypnotizing squares
Y	1	1	It makes us think of a infinite ravine
Y	8	4	It's a checkerboard
Y	1	2	It is very simple and not very beautiful
Y	10	1	It gives me the impression that it's round
Y	3	5	This painting is bizarre
Y	6	9	It looks like a chessboard that crashes a little in the middle
Y	6	5	It doesn't speak much to me. On the one side, it seems that it's a chessboard but more like made up of bands on a white line. It's weird
Y	8	2	I think this painting gives me a sense of depth
Y	3	5	We can see this painting as a book or a waterfall. The painting also changes when you fixate at certain points.
Y	5.75	2	It's a checkerboard that looks like an accordion. The impression of movement is more freeing than the previous one because you can imagine it moving towards the bottom or towards the top.
Y	2	0	It looks like a checkerboard that is infinitely falling.
Y	7	2	It's really strange, like a checkerboard that's falling.
Y	4	5	It's a chessboard that folds.
Y	9	4	It makes you think of a checkerboard
Y	5	0	A folding table
Y	5	0	Checkerboard
Y	6	5	It looks like there is a wall and then more to the right side of the wall there's some sort of a hallway. Also the 1st, 3rd, 5th, and so on look like they are coming out of the computer screen
Y	4	5	This painting is an optical illusion it moves as if it is like a book where you flip the pages

N	3	0	Very simplistic
N	6	3	A chess board made for very thin pawns
N	2	0	I don't know what to think about it
N	2	2	Unsymetrical squares that get smaller as they curve around a piller
N	4	0	The painting is very simple, with only squares. I did not see any movement though.
N	6	7	The painting is formed of squares at the left and right side which then become rectangles and meet at the middle right side. The squares are a black and white.
N	6	0	It reminds me of checkers and how I stopped playing them and started playing chess 4 years later
N	0	7	I think it looks like a race where cars will race and illusion
N	8	3	It looks like 2 sides are concaving in like folding and they get thinner in the middle)(<-- middle
N	8	2	Looks like a book folding, sort of looks like it moves from left to right
N	1	1	It hurts me
N	3	5	I don't like
N	8	1	I like it, it looks like it's going to suck you in and you'll get crushed by the squares
N	5	0	Less interesting
N	4	0	I could see what they were trying to do but it didn't work
N	7	3	Checkered pattern. When I focused I could see it become 3D like and the falling pattern bit became flat not like it looked when it was falling
N	5	1	Tiles are being absorbed reminds me of an art project I did in Grade 4 or 5
N	6	2	Checkered, contrast
N	5	3	Checkerboard falling in.
N	5	2	I like the rounded effect and also looking at the individual rows to observe how the rectangular shapes get thinner each time I move along the painting.

N	7	2	I think that these are squares of black and white colour, and that they have a limit but its not visible and it is hard to look right into the middle without getting confused.
N	2	3	It is weird
N	0.5	0	I find it interesting how the width of the rectangles gives me illusion of depth or they're being squeezed.
N	5	3	It seems like two walls that come close together or like two pages of a book that come together very closely in the end.
N	4	1	It is not attractive for me
N	7	0	It looks like a book, the pages of a book. It looks like the black squares could be anything. Asked if black squares were part of painting or if they weren't there
N	10	1	A depth part is hidden but can be noticed when you focus on it. I really like the idea though.
N	7	1	The painting makes me think of where it will eventually end up
N	5	1	It doesn't affect my eye
N	9	1	The checkers are going inward squishing into each other
N	7	2	When I was looking at this painting, it created images in my head. I thought of boxes, a cross, a book, empty space, also this painting looked like two things coming together
N	6	3	Looks like it's sinking in
N	2	1	The painting resembles a chessboard with a ravine in it. It seems like an optical illusion but I cannot see any movement
N	3	0	It's like a chessboard that has been heated from underneath and has melted inwards. Gives me a kinda "Alice in Wonderland" vibe. Also the optical illusion is well done, as it gives a very strong vertical movement to the painting.
N	5	0	This painting makes me think of a chessboard that is being fold
N	4	0	It is a checkered board that is moving inwards
N	5	5.5	Looks nice
N	2	0	I didn’t like how all the squares weren't the same size

N	3	1	It is very interesting but qutie simple, the artist just makes the white and black tiles thinner to make the painting appear as if it's going inwards
N	5	1	The painting is much like the Blaze but without the abrasive movement it creates
N	3	8	Quite simple, looks kind of like a book
N	9	1	It looks like a chessboard and it's like cloth that is waved to get rid of the dust or a slide in a waterpark. Like the chess figures will slide down with water and flight. Awesome
N	2	0	This painting did not make me see any optical illusion

Table S6: Painting 3

Dyslexic	Subjective Appreciation	Subjective destabilization	What do you think of the painting?
Y	7	2	It makes me think of sand and water.
Y	10	6	I think it's very beautiful with very beautiful colors.
Y	10	2	It's super beautiful.
Y	4	1	**It hurts my head**
Y	8	5	I really like this optical effect. The colors are pretty.
Y	8	7	There are waves and it moves. It's beautiful.
Y	4	1	It makes me think of the movement of water.
Y	4	5	It looks like waves that move from left to right.
Y	4	7	Gives the impression of waves that move.
Y	6	5	Interesting, I can see the optical illusion.
Y	10	10	It gives me the effect of waves on the water.
Y	7	10	It's hypnotizing.
Y	10	0	It makes me think of waves (and Pepsi with the colors).
Y	10	10	What's really funny also with the painting is that I don't see the optical illusion
Y	5	5	I like it a lot.
Y	9	8	Looks like waves.
Y	8	0	I like it a lot.
Y	9	9	Like I'm seeing waves
Y	1	5	Nothing.
Y	4	6	**The painting makes my eyes and head hurt if I look at it too long.**
Y	10	3	It's beautiful because it makes me think of the American flag.
Y	4	5	This painting has a lot of colors and I think it moves towards the left and to wards the right.
Y	7	6	Waves
Y	9	6	This painting moves me.
Y	6		It's beautiful with the waves.
Y	8	9	It's an ocean with color.
Y	4	7	It makes me think of an ocean with waves.
Y	10	0	I see waves striped and lined up.

Y	10	10	It's beautiful this image.
Y	10	4	Waves
Y	4	5	It seems like it will move like the ocean.
Y	9	9	There are waves, it's fascinating.
Y	7	9	It intrigues me and gives me an "optical illusion".
Y	10	1	It shows waves that move.
Y	3	4	It's just as strange as the other one.
Y	8	8	It looks like a wave.
Y	8	8	This one makes me feel more destablized.
Y	8	9	I think this painting is really troubling, because I see a lot of waves moving
Y	4	6	This painting is made of waves; when you fixate on them, they move like the ocean
Y	6	6	This is the most destabilizing painting and gives an impression of a flag in the wind or the undulating sea
Y	10	8	It looks like a moving sea. It makes me feel destabilized with waves that look very realistic
Y	9	4	It's a very nice painting, looks like a candy cane
Y	5	6	They are lines that transform while making waves
Y	10	0	It makes me think of a pink sea
Y	6	0	It looks like waves
Y	7	8	flag moving colorful
Y	7	8	It looks like waves from the sea or like there's a light breeze blowing over water and the water moves with the wind. Also at some times when I looked deeply at the painting I could see the blue and red mixing into purple
Y	10	10	This painting is like waves on an ocean of paper
N	9	5	very pleasing to the eye. Simple but complex
N	8	5	a very odd looking sea, perhaps on an alien planet, made of elongated colgate signs
N	7	4	looks like a wavy ocean
N	0	1	Makes me think of waves. They are warmer at the top and get colder as they come down
N	8	3	The painting was

N	9	10	The painting is blue, white, and red. It looks like a wave and some mountains. On the top right the colours have a higher contrast and when you move to the bottom-left the colours start to fade and mix colours
N	8	3	It reminds me of France because if you look from the side there you can see the French flag
N	0	9	an illusion with green/blue/red and white
N	1	10	I don't like this one mostly because it looks like waves or a flag moving with the wind but also the stripes are very annoying
N	9	6	I like that the center waves look like the aquafresh logo, very movemented, looks like its moving
N	0	0	it hurts me
N	10	7	I think about the painting very very beautiful
N	10	5	It's an awesome painting. It looked like colourful waves which moved all over the place.
N	9	5	very interesting; I like the wave effect and the colours
N	9	5	I like it, I like the choice of colors
N	10	3	it moved like waves, wave form
N	9	1	I love it. Looks like colourful sand dunes or waves. It's pleasing to look at and calming
N	8	5	wave-like, colorful
N	1	8	I hate it it makes me dizzi-ish
N	5	6	I like the waves and the illusion of fluidity of the painting. I don't like the red because it makes me uncomfortable and think of something negative. I like the blue color more.
N	9	5	I think that it is a flag of a country floating in the air and that it could make you feel calm
N	6	6	It reminds me of colgate
N	0	0.5	It looks like toothpaste. Also, the upper every line is placed slightly to the left to give the diagonal effect kinda cool
N	9	10	It looks like a blue, white, and red ocean that's moving
N	7	8	It's funny, it has a strange but cool feeling
N	10	0.5	It looks like painted current of water (the sea) colourful

N	6	2	It's very wavy. The wave aspect makes your brain think the image is moving though the image is very still.
N	9.5	7	This painting makes me think that it is moving because of the amount of curves
N	8	6	quite disturbing to the eye
N	9	0	I think it's really pretty and it reminds me of sand dunes
N	10	9	This painting looked like a wave, it reminded me of the sea, it also was moving (not stable).
N	6	9	It has the illusion that it is moving
N	8	7	The painting resembles a sea of toothpaste with waves moving across it. I find that the waves seem to move
N	5	0	It looks like the lovechild of a candy cane, a barbershop spiral, and a carpet company. Also, the individual stripe pattern reminds me of the Crest toothpaste logo.
N	5	1	this painting makes me think of candycane waves
N	6	6	this painting is an optical illusion that is a wave
N	7.5	8.5	I got lost in the image
N	6	1	It looks like a sea of toothpaste
N	5	4	It is the best optical illusion so far, I like the colours and the pattern is interesting
N	1	10	it's very interesting however I can't rate it highly if I genuinely feel uncomfortable while looking at it
N	4	7	looks like toothpaste
N	7.5	4	America! It's like an american sea or a beautiful carpet. I feel like jumping inside and swimming. Or like bumps in water parks. So pretty.
N	9	9	Very beautiful painting, reminds me of waves, the sea
